# Pneumococcal hydrogen peroxide regulates host cell kinase activity

**DOI:** 10.3389/fimmu.2024.1414195

**Published:** 2024-06-05

**Authors:** Jasmin Bazant, Astrid Weiss, Julia Baldauf, Ralph Theo Schermuly, Torsten Hain, Rudolf Lucas, Mobarak Abu Mraheil

**Affiliations:** ^1^ Institute of Medical Microbiology, German Centre for Infection Giessen-Marburg-Langen Site, Justus-Liebig University Giessen, Giessen, Germany; ^2^ Department of Internal Medicine, Cardio–Pulmonary Institute (CPI), Member of German Center for Lung Research (DZL), Justus-Liebig University Giessen, Giessen, Germany; ^3^ Vascular Biology Center, Medical College of Georgia at Augusta University, Augusta, GA, United States; ^4^ Department of Pharmacology and Toxicology, Medical College of Georgia at Augusta University, Augusta, GA, United States; ^5^ Division of Pulmonary, Sleep and Critical Care Medicine, Medical College of Georgia at Augusta University, Augusta, GA, United States

**Keywords:** *Streptococcus pneumoniae*, hydrogen peroxide, kinome analysis, Lck, Akt

## Abstract

**Introduction:**

Protein kinases are indispensable reversible molecular switches that adapt and control protein functions during cellular processes requiring rapid responses to internal and external events. Bacterial infections can affect kinase-mediated phosphorylation events, with consequences for both innate and adaptive immunity, through regulation of antigen presentation, pathogen recognition, cell invasiveness and phagocytosis. *Streptococcus pneumoniae* (*Spn*), a human respiratory tract pathogen and a major cause of community-acquired pneumoniae, affects phosphorylation-based signalling of several kinases, but the pneumococcal mediator(s) involved in this process remain elusive. In this study, we investigated the influence of pneumococcal H_2_O_2_ on the protein kinase activity of the human lung epithelial H441 cell line, a generally accepted model of alveolar epithelial cells.

**Methods:**

We performed kinome analysis using PamGene microarray chips and protein analysis in Western blotting in H441 lung cells infected with *Spn* wild type (*SpnWT*) or with *SpnΔlctOΔspxB* -a deletion mutant strongly attenuated in H_2_O_2_ production- to assess the impact of pneumococcal hydrogen peroxide (H_2_O_2_) on global protein kinase activity profiles.

**Results:**

Our kinome analysis provides direct evidence that kinase activity profiles in infected H441 cells significantly vary according to the levels of pneumococcal H_2_O_2_. A large number of kinases in H441 cells infected with *SpnWT* are significantly downregulated, whereas this no longer occurs in cells infected with the mutant *SpnΔlctOΔspxB* strain, which lacks H_2_O_2._ In particular, we describe for the first time H_2_O_2_-mediated downregulation of Protein kinase B (Akt1) and activation of lymphocyte-specific tyrosine protein kinase (Lck) via H_2_O_2_-mediated phosphorylation.

## 1 Introduction

The human genome encodes more than 535 protein kinases, which catalyse the phosphorylation of about 30% of all cellular proteins and function as reversible molecular switches, in order to regulate intracellular and extracellular signalling (1). These enzymes affect the functioning of the immune system and regulate transcription, metabolism, homeostasis, translation, cell cycle progression, differentiation, cytoskeletal rearrangement, apoptosis and intracellular communication (2, 3). Based on the specificity for their downstream targets, protein kinases can be separated into different groups: 1) protein tyrosine kinases (PTKs), 2) serine/threonine kinases (STKs), 3) dual specificity kinases, which phosphorylate both serine/threonine and tyrosine residues and 4) histidyl kinases, which transfer the phosphate group onto an aspartate residue (4). The majority of the human protein kinases -428- belong to the STKs, whereas 90 are PTKs (5).

Infections of mammalian cells with pathogenic bacteria can affect phosphorylation-based signalling (6). According to the WHO, infections with *S. pneumoniae* (*Spn*) are responsible for 1.6 million deaths worldwide each year, including around 700,000 deaths in children below 5 years of age (7). *Spn* is an encapsulated Gram-positive pathogen that was found to reduce the activity of certain protein kinases such as Adenosine Monophosphate-activated Protein Kinase (AMPK-α) and Cyclin-dependent kinase (CDK) in infected mice (8). *Spn-*infection of THP-1 cells can reduce the activity of protein kinase B (a.k.a. Akt kinase), involved in phagocytosis (9). As such, the pathogen can modify the host’s kinome to promote its survival. *Spn* possesses several virulence factors, including capsule and adhesion proteins (10), as well as the cholesterol-dependent cytolysin pneumolysin (Ply) and hydrogen peroxide (H_2_O_2_). Endogenously generated H_2_O_2_ is a by-product of the enzymes pyruvate oxidase (SpxB) and lactate oxidase (LctO) in *Spn* (11). Whereas SpxB converts pyruvate, inorganic phosphate and oxygen into acetyl phosphate, carbon dioxide (CO_2_) and H_2_O_2_ (12, 13), LctO catalyses the formation of pyruvate and H_2_O_2_ from L-lactate and oxygen (11, 14). Whereas physiological H_2_O_2_ levels in human plasma are in the range of 1 to 8 µM (15), *Spn*-derived H_2_O_2_ can surpass concentrations of 1 mM in culture supernatants (16). The endogenous production of high levels of H_2_O_2_ by *Spn* provides protection against other commensals like *Staphylococcus aureus, Haemophilus influenzae, Moraxella catarrhalis* and *Neisseria meningitidis* during colonisation of the nasopharynx (17). Moreover, pneumococcal-derived H_2_O_2_ is considered as a virulence factor and induces DNA double-strand breaks and apoptosis in human alveolar epithelial cells and impairment of the alveolar-capillary barriers, both of which promote the spread of *Spn* in the host (18–20). Interestingly, deletion of SpxB decreases virulence of *Spn in vivo* (21).

To date, it is not known what pneumococcal virulence factor, if any, is the main mediator of *Spn*-induced kinase regulation in mammalian cells. In this study, we investigated the influence of pneumococcal H_2_O_2_ on the protein kinase activity of the H441 human lung epithelial cell line, a generally accepted model of alveolar epithelial cells (22). The effects of H_2_O_2_ released by *Spn* during infection on the (de-) activation status of the global kinome were investigated using a peptide-based kinase activity assay (PamStation platform), which enables identification of differentially activated kinases based on the phosphorylation of immobilized substrate peptides in infected lung cells (23). In our approach, we compared the kinomes of H441 cells infected with either *Spn* wild type (*SpnWT*) or *Spn*Δ*lctO*Δ*spxB* mutant, the latter of which lacks LctO and SpxB and is therefore strongly attenuated in H_2_O_2_ production (generates only 3% of *SpnWT* H_2_O_2_ levels) (11). As we and others have previously shown that the *Spn*Δ*lctO*Δ*spxB* mutant releases significantly lower amounts of the pore-forming toxin Ply, as compared to *SpnWT* (24, 25), we also analyzed the kinome of cells infected with the Ply deletion mutant *SpnΔply*, in order to exclude specific effects caused by reduced release of Ply in infected cells.

Our data demonstrate that kinase activity profiles in infected H441 cells significantly vary according to the levels of pneumococcal H_2_O_2_. Infection with *SpnWT* prominently downregulated several protein kinases of both the STK and PTK groups. These kinases, including protein kinase B (Akt), were significantly downregulated in *SpnWT*-infected cells, but not in *Spn*Δ*lctO*Δ*spxB-*infected cells.

By contrast, Activity of Lymphocyte-specific tyrosine protein kinase (Lck) was significantly higher in cells infected with the *Spn*Δ*lctO*Δ*spxB* mutant than in cells infected with the WT strain, in view of an H_2_O_2_-dependent increase in inhibitory phosphorylation of Lck at residue tyrosine 505. As a proof of principle, we demonstrate that the phosphorylation of both Akt (leading to its activation) and Lck (resulting in its inhibition) is affected by the addition of exogenous H_2_O_2_.

## 2 Materials and methods

### 2.1 *Bacterial strain* and growth conditions

*Streptococcus pneumoniae* wild type (*SpnWT*) D39 (Serotyp 2), deletion mutants *Spn*Δ*ply* (Serotyp 2) (kindly provided from Prof. Hammerschmidt, University Greifswald) and *Spn*Δ*lctO*Δ*spxB* (Serotyp 2) (kindly provided from Prof. Winkler and Prof. Giedroc, University of Indiana) were freshly plated on blood agar plates from cryocultures (stored at -80°C). Blood agar plates were incubated for 15-17 hours (h) at 33°C/5% CO_2_. *Spn* cultures with an OD_600nm_ of 0.04 were grown in Todd-Hewitt broth medium (BD) with 0.5% yeast extract (THB-Y) and incubated at 37°C/5% CO_2_.

### 2.2 Cell culture conditions

Human lung adenocarcinoma cell line NCI-H441 (H441) was cultured in Roswell Park Memorial Institute (RPMI) 1640 medium (Gibco) with 10% heat-inactivated fetal calf serum (FCS) (Superior, Thermo Fisher) at 37°C with 5% CO_2_.

### 2.3 Infection assays and cell lysis

H441 cells were seeded in 10 cm cell culture plates at a density of 1.8×10^6^ cells/plat and incubated at 37°C with 5% CO_2_ for 48 h until 90% confluency. *Spn* cultures were grown on the day of infection in THB-Y medium for 3 h incubated at 37°C with 5% CO_2_ to an OD_600nm_ of 0.40 and washed 2x with PBS and centrifuged for 4 min at 6,000 g. *Spn* pellets were resuspended in RPMI medium supplemented with 10% FCS. *SpnWT* or *Spn*Δ*ply* or *Spn*Δ*lctO*Δ*spxB* suspensions were adjusted to a multiplicity of infection (MOI) 45 in infection medium and added to H441 cells for 5 h. Then the cell culture supernatants were removed, and the remaining H441 cells were washed with 5 mL ice cold PBS. Finally, the cells were scraped down in 250 µl M-PER mammalian extract buffer (Thermo Scientific) containing protease inhibitor cocktail (Thermo Scientific) and Halt phosphatase inhibitor (Thermo Fisher Scientific). Next, samples were homogenised with a syringe and incubated for 1 h at 4°C followed by centrifugation for 15 min at 16,000 g at 4°C. The supernatants were aliquoted and frozen in liquid nitrogen and stored at -80°C. Individual aliquots were used for protein concentration determination (BCA assay), Western blot (Jess Simple Western) or peptide-based kinase activity (PamStation) measurement.

### 2.4 PamStation kinome analysis

H441 cells infected with *SpnWT* or *Spn*Δ*ply* or *Spn*Δ*lctO*Δ*spxB* cultures were lysed as described in 2.3 and subjected to kinome profiling as described previously (26–28). For each condition, five experimental replicates were performed and non-infected cells were used as control group. For PTK analyses, 10 µg of the protein lysate (2 µg for STK, respectively) were dissolved in reaction buffer (proprietary information by the manufacturer) and added on the PamChip for analysis of tyrosine kinases (or serine/threonine, respectively). Peptide substrate phosphorylation was detected with secondary FITC-conjugated antibodies and monitored by a CCD camera (Evolve (Software Version), PamGene). All recorded images were further processed by bioinformatic software to derive a single numerical value reflecting the intensity of phosphorylation for each peptide and each sample (BioNavigator6, Version 6.3.67.0, PamGene). Individual values were corrected by logarithmical transformation and mathematical normalisation for the different replicates (x_center_ = x – mean[x]) before subsequent processing for database-assisted upstream kinase prediction. Kinases responsible for significant changes in peptide phosphorylation between two experimental conditions (e.g. non-infected control versus *SpnWT*), are described by two important parameters: The predicted differential kinase activity depicts the overall change of the peptide set that represents the group of substrates for the given kinase. In addition to this, the “mean specificity score” is stated, which is expressed as the negative log_10_ p-value, where p < 0.05 refers to the statistical significance for the changes of the phosphorylation for the substrate peptide sets between the two experimental conditions. Therefore, kinases with a prediction of a highly differential kinase activity and a “mean specificity score” higher than 1.3 were considered as promising candidates for subsequent investigations. The step-wise comparisons of two distinct experimental groups facilitated the identification of *Spn* mutant specific peptide phosphorylation and the activation of responsible upstream kinases by Venn diagram analyses (BioVenn - a web application for the comparison and visualization of biological lists using area-proportional Venn diagrams (29).

### 2.5 Automated western immunoblotting with Jess™ simple western

Jess Simple Western system (ProteinSimple, San Jose CA, USA) is an automated capillary-based size separation immunoassay. This technology was used to detect protein levels of kinases in lysates from H441 cells according to the manufacturer’s instructions. For detection of Akt and Lck levels anti-p-Akt antibody (Cell Signaling 9271), anti-Akt antibody (Cell Signaling 9272), anti-Lck antibody (Cell Signaling) and anti-p-Lck antibody (Cell Signaling 2751) were used. Briefly, 2.4 µl protein extract was mixed with 0.6 µl fluorescent 5x master mix supplied 200 mM dithiothreitol (DTT) dissolved in sample buffer (Protein simple). All samples were denatured at 95°C for 5 min. Afterwards, the samples were spinned down and together with the size ladder (12-230 kDa) placed on ice until loading onto the plate. Once the plate is centrifuged (1,000 g, 5 min, room temperature) and placed into the instrument together with the cartridge, a fully automated process with standard instrument settings ensures separation of the samples followed by immobilization via UV light and chemiluminescent detection using primary and secondary horse-radish peroxidase antibodies. Images, i.e. light emission, are recorded by a CCD camera and analysis is performed by the Compass Simple Western software (version 4.1.0, Protein Simple). The results, i.e. protein expression reflected by band intensities, are displayed in traditional lane view as well as electropherograms, which allow for quantification by defining the respective area under the curve.

### 2.6 Statistical analyses

Statistical analyses were carried out using one-way ANOVA with GraphPad Prism software 5 (GraphPad Software, Inc., La Jolla, CA, USA). p < 0.05 was considered to indicate a statistically significant difference.

## 3 Results

### 3.1 Pneumococcal H_2_O_2_ downregulates kinase activity in Spn-infected H441 cells

**Figure 1:**
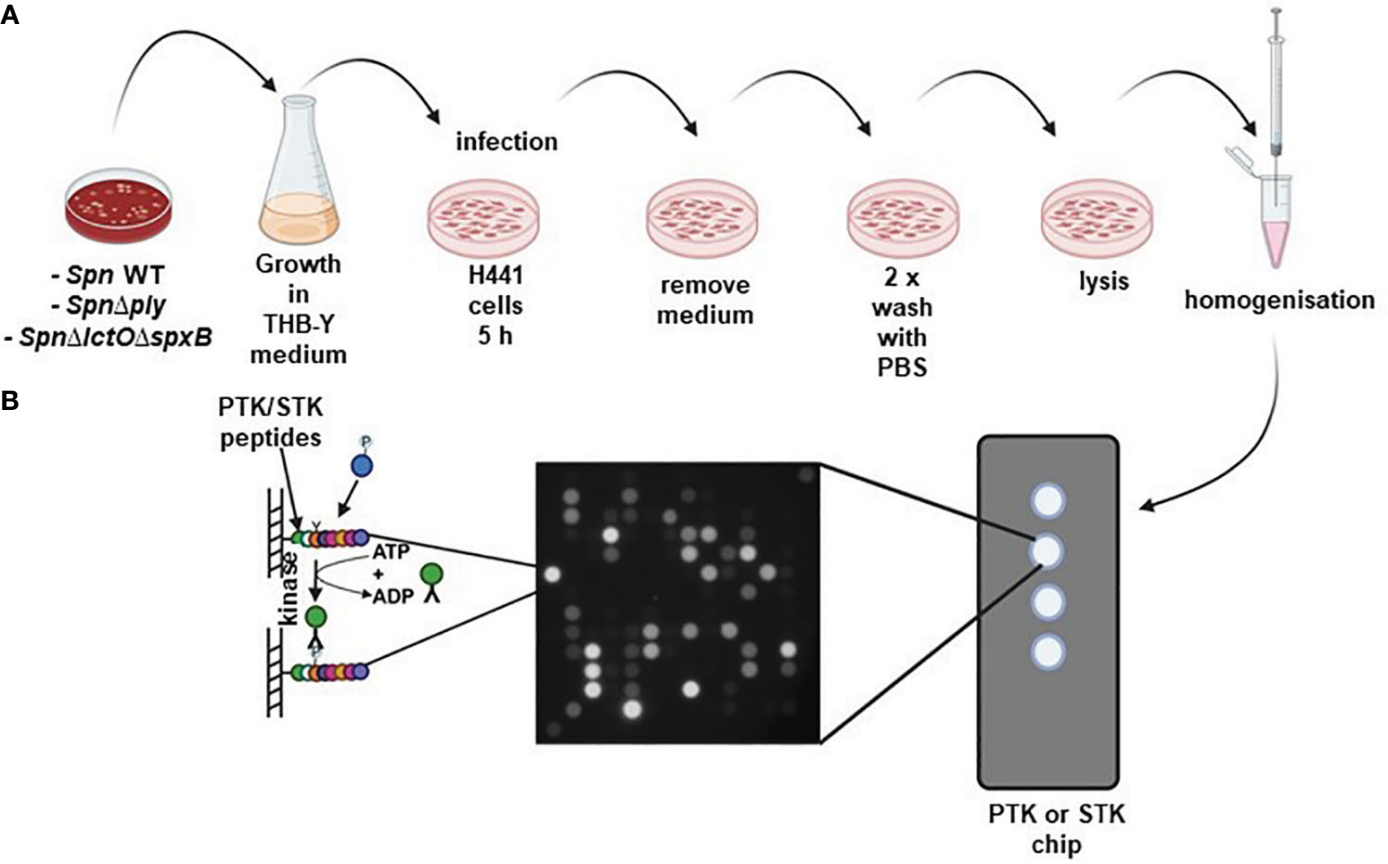
**(A)** Schematic representation of *Spn* infection: H441 lung cells were separately infected with either *SpnWT*, *Spn*Δ*lctO*Δ*spxB* or *Spn*Δ*ply* for 5 (h) Non-infected vehicle-treated H441 cells were used as control. **(B)** Kinome profiling of *Spn-*infected H441 lung cells using PamStation assay. Cell lysates were placed on protein tyrosine kinase (PTK) and serine/threonine kinase (STK) PamStation Chips. Every Chip contains four arrays covered with covalently bound peptides. Activated kinases present in the cell lysates phosphorylate peptides on the Chip. Phosphorylation of PTK or STK substrate peptides was detected using anti-phosphorylation antibodies and secondary FITC-conjugated antibodies. Created with BioRender.

In order to investigate whether *Spn-*derived H_2_O_2_ affects protein kinase activity in lung cells, we infected H441 lung cells with *SpnWT* or with a deletion mutant strongly impaired in H_2_O_2_ production, due to the lack of pyruvate oxidase (SpxB) and lactate oxidase (LctO): *Spn*Δ*lctO*Δ*spxB* (11). Additionally, a pneumolysin (Ply) deletion mutant (*Spn*Δ*ply*) was tested, to exclude effects caused by the decreased release of Ply observed in the *Spn*Δ*lctO*Δ*spxB* mutant (24, 25). Non-infected vehicle-treated or *Spn*-infected H441 cells were lysed for protein isolation five hours (h) post treatment/infection, respectively. Kinome profiling of lysates from infected or non-infected cells was performed on microarray chips using the PamStation platform to analyze kinase activities of protein tyrosine kinases (PTKs) and serine/threonine kinases (STKs) (**Figures 1 A, B**). PamGene microarray chips contain designed substrate peptides, representing kinase phosphorylation sites for PTKs (196 different peptides in total) or STKs (144 different peptides in total) (30).

**Figure 2:**
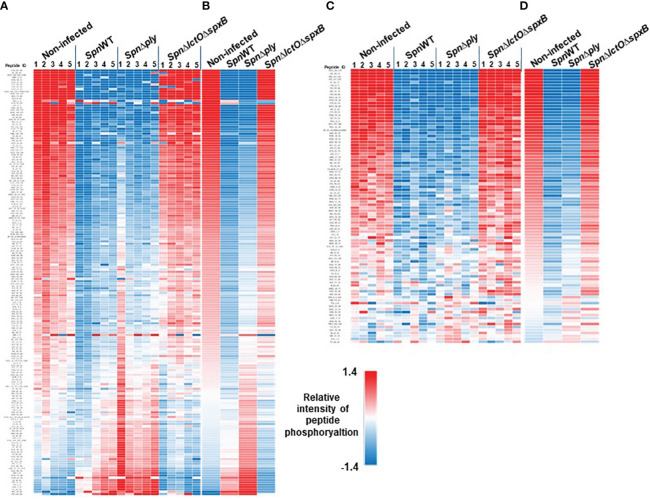
Intensities of peptide phosphorylation induced by differential kinase activities in non-infected H441 cells and H441 cells infected with *SpnWT*, *Spn*Δ*lctO*Δ*spxB* or *Spn*Δ*ply*. The heat maps illustrate the intensities of peptide phosphorylation of five independent experiments. Relative intensities of peptide phosphorylation of protein tyrosine kinases (PTKs) **(A)** and serine/threonine kinases (STKs) **(C)** are depicted. **(B, D)** represent the means of all determined relative intensities of PTK and STK peptide phosphorylation.

The differential pattern of kinase-mediated peptide-phosphorylation in protein extracts of H441 cells infected with different *Spn* strains (*SpnWT*, *Spn*Δ*lctO*Δ*spxB* or *Spn*Δ*ply*) vs. non-infected cells from five biological experiments and their respective mathematical mean values are visualized as heat maps in **Figure 2** (**Figures 2A, B** for PTK, **Figures 2C, D** for STK). The figure depicts relative changes in phosphorylation of the highly dysregulated peptides on the PTK PamChips (182 peptides in total after quality control) and STK PamChips (92 peptides in total after quality control) representing kinase substrate phospho-sites.

Peptide phosphorylation patterns for PTKs as well as STKs in non-infected H441 cells and those infected with *Spn*Δ*lctO*Δ*spxB* indicate more similarities between each other as compared to the very different patterns of non-infected controls on the one hand and cells infected with *SpnWT* or *Spn*Δ*ply* on the other hand, the latter two of which generate high levels of H_2_O_2_. Interestingly, infections with *SpnWT* and *Spn*Δ*ply* show a similar pattern, indicating that the lack of Ply does not greatly affect the kinome (**Figures 2A–D**). The kinases (both STK and PTK) are mainly downregulated in *SpnWT*- and *Spn*Δ*ply-*infected cells, as compared to *Spn*Δ*lctO*Δ*spxB-*infected cells. Taken together, these results demonstrate an important effect of *Spn*-derived H_2_O_2_ on human kinase activity in H441 cells. The full data sets representing the underlying numerical values of the phosphorylation intensities are listed in **Supplementary Tables S1**, **S2** (**Supplementary material**).

**Figure 3:**
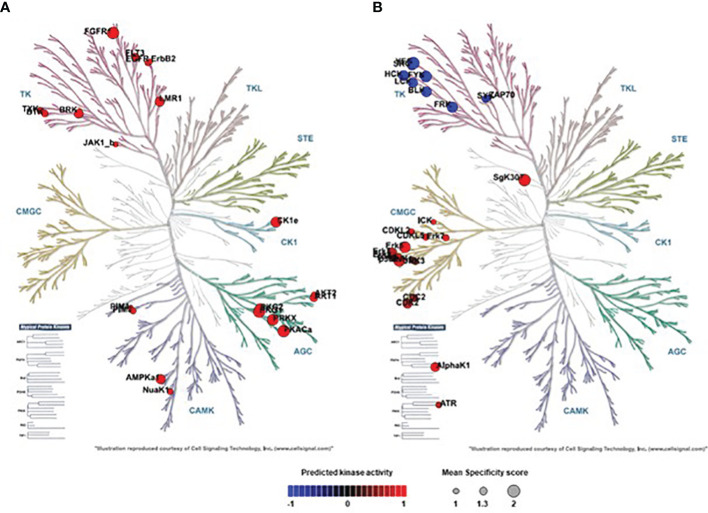
Phylogenetic mapping of top-ranked dysregulated Serine/threonine kinases (STKs) and Protein-Tyrosine kinases (PTKs) in *SpnWT-* and *Spn*Δ*lctO*Δ*spxB*-infected H441 cells. **(A)** *SpnΔlctOΔspxB* vs. *SpnWT*: Phylogenetic kinome tree shows distribution of kinases analysed in the activity profiles of PTKs and STKs of cells infected with *SpnWT* vs. *Spn*Δ*lctO*Δ*spxB* cluster in Tyrosine Kinase (TK) family (Brk, Lmr1, FGFR1, FLT3) in AGC family (Akt1, PKG1, PKG2, PKAα, PRKX) and CAMK family (AMPKα1) and in CK1 family (CK1). **(B)** *SpnΔlctOΔspxB* vs. non-infected: Phylogenetic kinome tree illustrates distribution of kinases analysed in the activity profiles of PTKs and STKs of cells infected with *Spn*Δ*lctO*Δ*spxB* compared to control cells. Significantly dysregulated kinases cluster in TK family (FRK, Src, Fyn, Blk, Lck, Hck, Yes) and CMGC kinase group (CDK1, CDK2, ERK1, ERK2, ERK5, p38-δ, p38-γ, AlphaK1, SgK307). Circle size of activated kinases in phylogenetic kinome trees were based on Specificity score (1 to 2). The default was set to colouring according to Normalized kinase statistic (s). The colouring scale shows changes in kinase activity ranges from −1 (blue = indicates decrease of kinase activity) to +1 (red = indicates increase of kinase activity). P-value < 0.05.

Based on the determined phospho-peptide signatures comparing two experimental groups, a bioinformatics-based analysis of upstream kinases was performed, allowing for a prediction of kinase activity that is differentially regulated between the depicted conditions. Phylogenetic mapping of top-ranked dysregulated kinases (specificity score > 1.3) in H441 cells infected with *Spn*Δ*lctO*Δ*spxB* vs. *SpnWT* revealed a kinase cluster that was not downregulated in *Spn*Δ*lctO*Δ*spxB*: i) tyrosine kinase (TK) family [Lemur tyrosine kinase 1 (Lmr1), Fibroblast growth factor receptor 1 (FGFR1), FMS-like tyrosine kinase 3 (FLT3), Breast tumor kinase (Brk)] ii) AGC family [cGMP-dependent protein kinase 1 (PKG1, PKG2), Protein kinase A α (PKAα), Protein kinase X (PRKX), protein kinase B (a.k.a. Akt kinase)] iii) calcium/calmodulin-dependent protein kinase (CAMK) family [AMP‐activated protein kinase α1 (AMPKα1)] iv) CK1 family casein kinase 1 (CK1)] (**Figure 3A**).

The comparison of *Spn*Δ*lctO*Δ*spxB* vs. non-infected cells unveiled some moderately dysregulated kinases. Seven kinases belonging to TK family [Fyn-related kinase (FRK), Src proto-oncogene kinase (Src), Fyn proto-oncogene (Fyn), B lymphoid kinase (BLK), Lymphocyte-specific tyrosine protein kinase (Lck), Hematopoietic cell kinase (Hck), Yes proto-oncogene (Yes)] are downregulated. Additionally, several members of CMGC kinase group including Cyclin-dependent kinase 1 (CDK1), CDK2, Extracellular-signal Regulated Kinase1 (ERK1), ERK2, ERK5, p38-δ, p38-γ, and Alpha-Kinase 1 (AlphaK1), SgK307 are moderately but significantly upregulated after infection with *Spn*Δ*lctO*Δ*spxB* compared to non-infected cells (**Figure 3B**).

### 3.2 H_2_O_2_-generating *SpnWT and SpnΔply* decrease phosphorylation of Akt1 kinase

**Figure 4:**
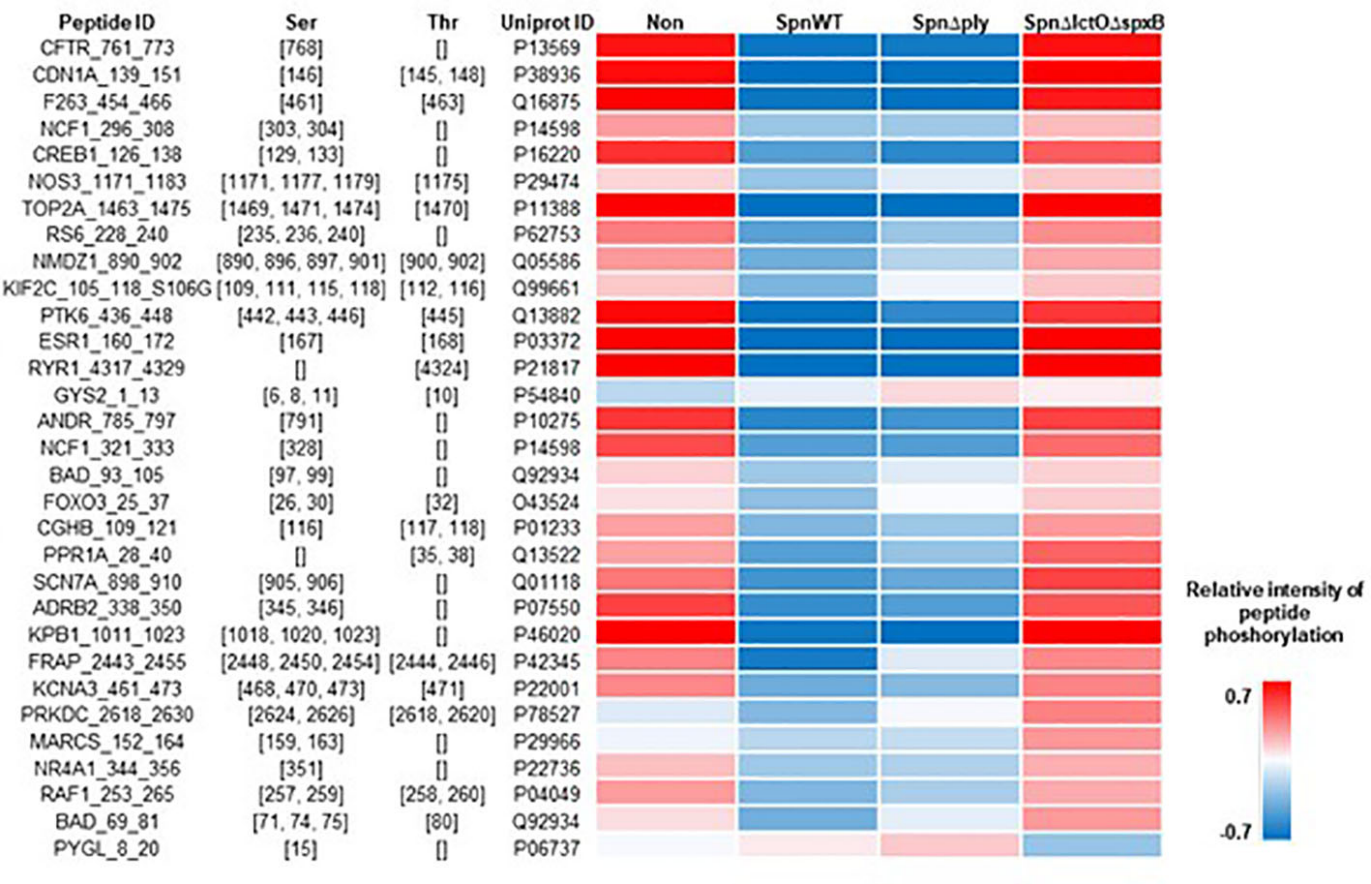
Intensity of phosphorylation of distinct peptides that serve as substrates for Akt1 kinase in PamStation assays. Peptide ID with sequence information together with the respective phospho-site, i.e. Ser or Thr residues, are given. The color-coded heat map displays the relative intensitiy of phosphorylation after normalization of the log-transformed raw data. For all four experimental groups, i.e. non-infected, *SpnWT*, *SpnΔply*, and *SpnΔlctOΔspxB*, the respective sets of five replicates each were used to calculate the final value, i.e. mathematical mean.

Protein kinase B (Akt1) belongs to significantly upregulated kinases in *Spn*Δ*lctO*Δ*spxB* (**Figure 3A**). Akt1 is involved in cell cycle progression, cell growth, cell survival and apoptosis and is part of the highly conserved phosphatidylinositol-3 kinase (PI3K)/Akt/mammalian target of rapamycin (mTOR) signaling pathway (26, 27). The phosphorylation of immobilized peptides that serve as substrates for Akt1 in the PamStation assays are depicted in **Figure 4**. The relative intensities of peptide phosphorylation that serve as Akt1 substrates demonstrates significant downregulation of profiles in cells infected with *SpnWT* and *SpnΔply* (H_2_O_2_ producers) (**Figure 4**). In contrast, the peptide phosphorylation profiles of cells infected with *SpnΔlctOΔspxB* and non-infected cells show a significantly higher activity of Akt1, as compared to cells infected with *SpnWT* (**Figure 4**).

**Figure 5:**
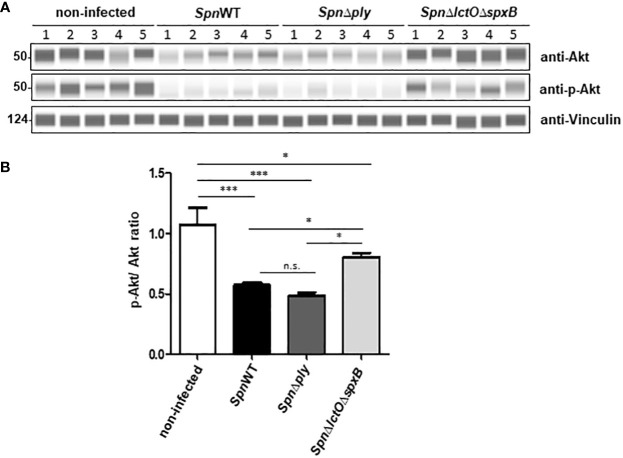
*Spn*-derived H_2_O_2_ reduces p-Akt/Akt ratio in H441 cells. **(A)** Protein levels of total Akt and phosphorylated Akt were analysed by Western blot after infection of H441 cells with *SpnWT*, *Spn*Δ*ply* and *SpnΔlctO*Δ*spxB*. Five independent infections were performed and lysates were analysed with anti-p-Akt, anti-Akt and anti-Vincullin antibody. **(B)** Quantification of p-Akt/Akt ratios following determination of protein levels of phosphorylated Akt and total Akt (p-Akt/Akt) by Western blot. Significant differences are denoted with asterisks (p < 0.05 = *; p < 0.001 = ***). n.s., not significant.

In order to confirm the effect of endogenously generated pneumococcal H_2_O_2_ on Akt kinase activity, we performed Western blot analysis using specific antibodies against total and phospho-Akt to measure their ratio in H441 lung cells infected with *SpnWT*, *Spn*Δ*ply* and *Spn*Δ*lctO*Δ*spxB*. The results show that infection of H441 cells with *SpnWT* and *Spn*Δ*ply* decreases both Akt total protein expression and phosphorylation, assessed as the p-Akt/Akt ratio (**Figures 5A, B**). Akt levels and p-Akt/Akt ratio in *Spn*Δ*lctO*Δ*spxB*-infected cells as well as in non-infected cells are significantly higher than in *SpnWT*-infected cells (**Figures 5A, B**). The reduced p-Akt/Akt ratio in *SpnWT-*infected cells, as compared to infection with *Spn*Δ*lctO*Δ*spxB* is consistent with the predicted activation of Akt in H441 lung cells detected by PamStation kinome profiling data (**Figures 3A**, **4**). Reduction of p-Akt/Akt ratio in H441 cells after infection with H_2_O_2_ generating strains (*SpnWT* and *Spn*Δ*ply*), but not *Spn*Δ*lctO*Δ*spxB* strongly suggests an effect of H_2_O_2_ on phosphorylation of Akt.

### 3.3 Enhanced inhibitory Lck phosphorylation in the absence of H_2_O_2_

**Figure 6:**
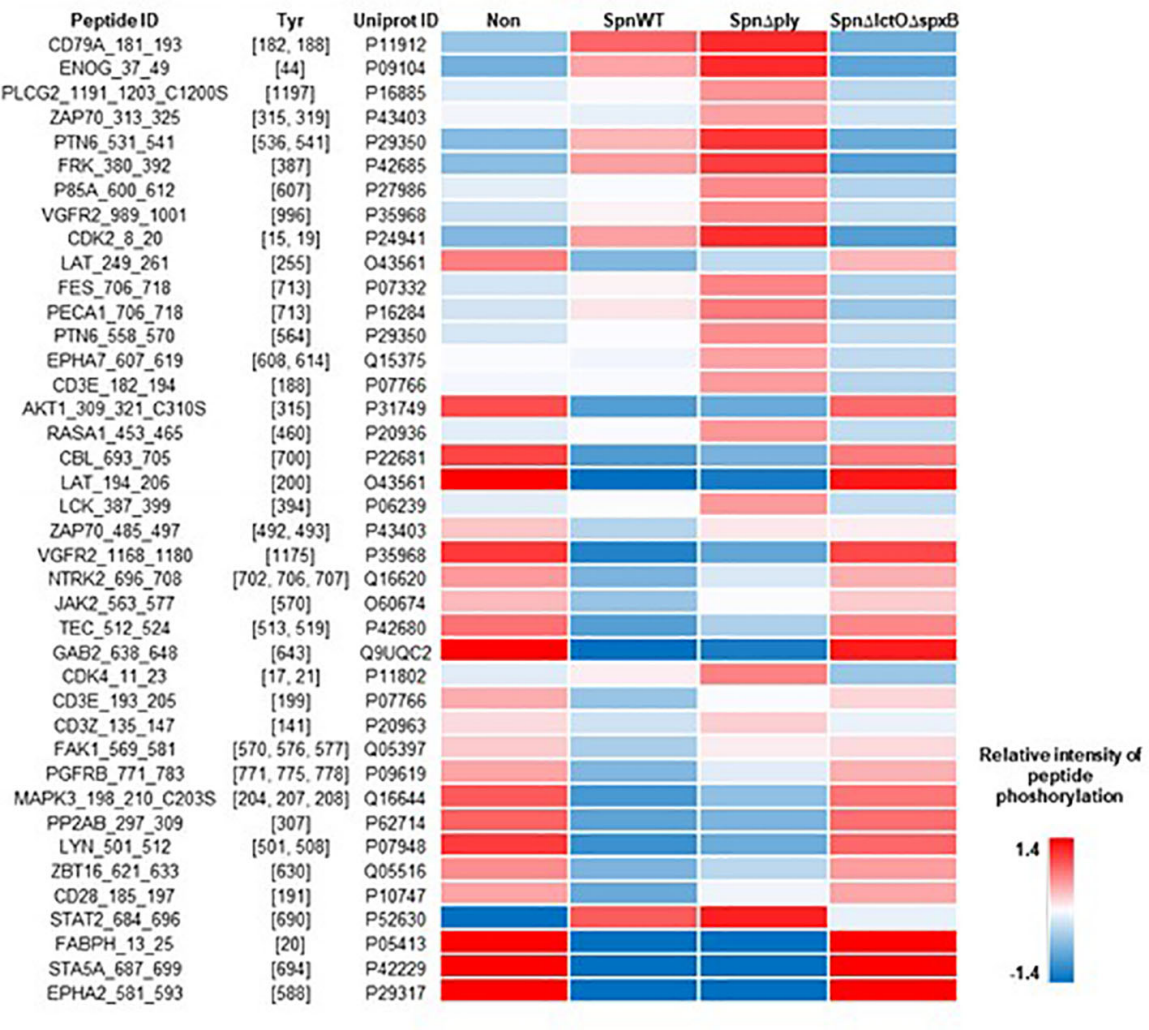
Intensity of phosphorylation of distinct peptides that serve as substrates for the Lck kinase in PamStation assays. Peptide ID with sequence information together with the respective phospho-site, i.e. Tyr residues, are given. The color-coded heat map displays the relative intensity of phosphorylation after normalization of the log-transformed raw data. For all four experimental groups, i.e. non-infected, *SpnWT*, *SpnΔply*, and *SpnΔlctOΔspxB*, the respective sets of five replicates each, were used to calculate the final value, i.e. mathematical mean.

The lymphocyte-specific tyrosine protein kinase (Lck) plays a role in cell proliferation and apoptosis of H441 cells, used in this study (31). Lck catalytic activity was reported to be induced by H_2_O_2_ (32, 33). Phosphorylation of Tyr-505, located near the carboxyl terminus of Lck, was reported to induce inhibition of Lck, due to stabilisation of a biologically inactive conformation (34). The consequences of H_2_O_2_ production by *SpnWT* and *SpnΔply* on the phosphorylation intensities of immobilized peptides that serve as substrates for Lck in PamStation assays are illustrated in **Figure 6**. The results show similar phosphorylation patterns between cells infected with the H_2_O_2_-producing strains *SpnWT* and *SpnΔply*. On the other hand, non-infected or *SpnΔlctOΔspxB-*infected cells indicate phosphorylation patterns similar to one another but very different from *SpnWT*-infected cells (**Figure 6**).

**Figure 7:**
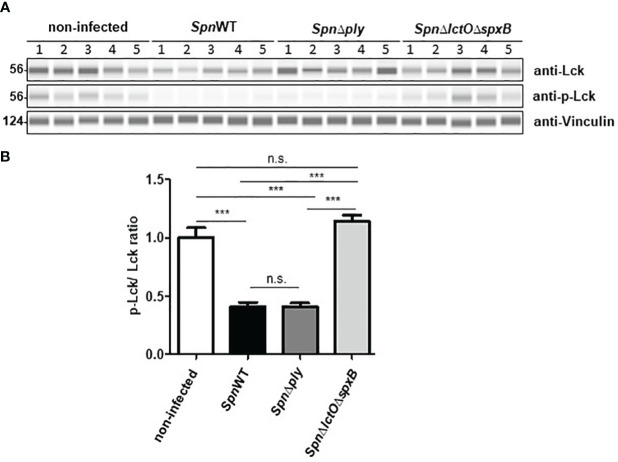
H_2_O_2_ generating *Spn* strains (*SpnWT*, *Spn*Δ*ply*) reduce p-Lck/Lck ratio. **(A)** Protein levels of total Lck and phosphorylated Lck were analysed by Western blot after infection with *SpnWT*, *Spn*Δ*ply* and *SpnΔlctO*Δ*spxB*. Five independent infections were performed and lysates were analysed with anti-p-Lck (Y505), anti-Lck and anti-Vincullin antibody. **(B)** Diagram of p-Lck/Lck ratio illustrated protein levels of phosphorylated Lck to total Lck (p-Lck/Lck) detected by Western blot. Significant differences are denoted with asterisks (p < 0.001 = ***). n. s., not significant.

In order to analyse the effect of pneumococcal H_2_O_2_ on Lck activity in H441 cells infected with *SpnWT*, *Spn*Δ*ply* and *Spn*Δ*lctO*Δ*spxB*, Lck and p-Lck levels were measured using anti-Lck antibody and anti-phospho Tyr505-Lck antibody to detect alterations in the phosphorylation of Lck during infection. The results show inactivation of Lck in H441 cells infected with *Spn*Δ*lctO*Δ*spxB* and non-infected cells, as determined by increased phosphorylation of Lck at tyrosine 505 accompanied by a comparable high p-Lck/Lck ratio (**Figures 7A, B**). Interestingly, H_2_O_2_-generating strains (*SpnWT* and *Spn*Δ*ply*) seem to maintain or even induce the Lck activity indicated by decreased phosphorylation of Lck at tyrosine 505 leading to a lower p-Lck/Lck ratio. These finding suggest H_2_O_2_-mediated activation of Lck.

### 3.4 Addition of external H_2_O_2_ modulates kinase activity of Lck and Akt

**Figure 8:**
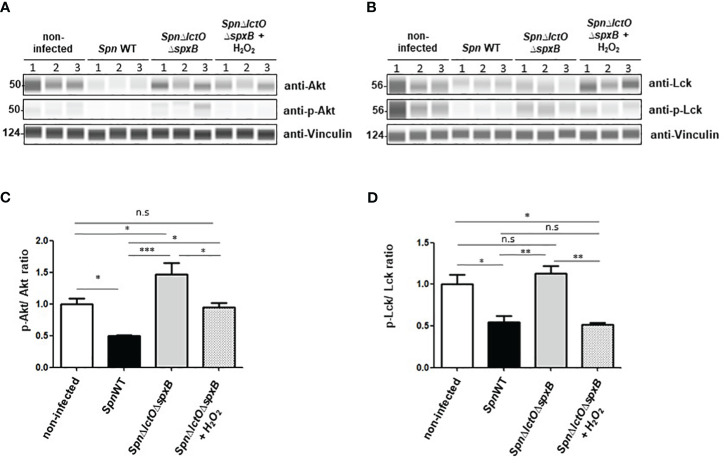
Addition of external H_2_O_2_ modulates kinase activity of Akt and Lck. **(A, B)** Phosphorylation of Akt and Lck were analysed by Western blot in cells infected with *SpnWT*, *Spn*Δ*ply*, *SpnΔlctO*Δ*spxB* or with *SpnΔlctO*Δ*spxB* in the presence of exogenous H_2_O_2_ (1 mM). H441 cell lysates of three biological experiments (1, 2 and 3) were analysed with anti-p-Akt, anti-Akt, anti-p-Lck (Tyr-505), anti-Lck and anti-Vincullin antibody. **(C)** Quantification of p-Akt/Akt ratio following determination of protein levels of phosphorylated Akt to total Akt (p-Akt/Akt) detected by Western blot. **(D)** Diagram of p-Lck/Lck ratio shows protein levels of phosphorylated Lck to total Lck (p-Lck/Lck) detected by Western blot. Significant differences are denoted with asterisks (p < 0.05=*; p < 0.01 = **; p < 0.001 = ***). n. s., not significant.

In order to substantiate that the detected differences in Lck and Akt activities in *SpnWT-*infected vs. *Spn*Δ*lctO*Δ*spxB*-infected H441 cells are predominantly due to pneumococcal H_2_O_2_ (**Figures 5**, **7**), we performed infection experiments in the presence of exogenously added H_2_O_2_ (1 mM) in cells infected with *Spn*Δ*lctO*Δ*spxB*. As such, we found that the infection with *SpnWT* as well as addition of exogenous H_2_O_2_ to cells infected with *Spn*Δ*lctO*Δ*spxB* significantly reduced the levels of p-Akt and non-phosphorylated Akt as compared to *Spn*Δ*lctO*Δ*spxB-*infected cells (**Figures 8A–C**). Levels of Lck phosphorylation were reduced in H441 cells infected with *SpnWT* or with *Spn*Δ*lctO*Δ*spxB* after additional H_2_O_2_ treatment (**Figures 8B–D**). The reduction of Lck and Akt phosphorylation levels triggered by exogenous H_2_O_2_ treatment of *Spn*Δ*lctO*Δ*spxB-*infected cells was similar to what was observed following *SpnWT* infection, indicating a direct impact of pneumococcal-derived H_2_O_2_ on the phosphorylation and activity of Akt and Lck.

## 4 Discussion

Protein kinase-mediated phosphorylation represents the most common post-translational protein modification in signalling processes. Kinase phosphorylation events triggered by bacterial infections can have consequences for the immune response by regulation of antigen presentation, pathogen recognition, cell invasiveness and phagocytosis (6, 9). Infection of mice with *Spn* was previously demonstrated to reduce the activity of Cyclin-dependent kinase (CDK) and Adenosine Monophosphate-activated Protein Kinase (AMPK-α) (8). Protein kinase B (a.k.a. Akt) was shown to promote phagocytosis of *Spn* by THP-1 macrophages (9). Since the role of the pneumococcal virulence factor H_2_O_2_ in potential changes to the host’s kinome has not been investigated in detail, in this study, we focused on the role of endogenously generated H_2_O_2_ in changes of the host’s kinome following *Spn* infection. Pneumococcal H_2_O_2_ provides not only an advantage against other competitive commensals, but can also lead to cell damage, DNA damage, and apoptosis in human alveolar lung epithelial cells (17–20). H_2_O_2_ can also foster intracellular *Spn* survival by oxidative inactivation of the host’s lysosomal cysteine cathepsins (35). H_2_O_2_ has the capacity to modulate protein kinase activity either directly by oxidation of kinase regulatory cysteine and/or methionine residues or indirectly by oxidation of protein tyrosine phosphatases (PTPs) (36–38). In agreement with our results showing a significant downregulation of Akt activity following infection by *SpnWT*, but not by mutant *Spn*Δ*lctO*Δ*spxB*, oxidation of regulatory cysteine residues by H_2_O_2_ was shown to inactivate Akt kinase and Fibroblast growth factor receptor (FGFR) kinase (39, 40). H_2_O_2_ is also able to indirectly modulate activity of certain tyrosine kinases -such as focal adhesion kinase (FAK)- through inhibition of tyrosine phosphatase (38, 41). In this regard, oxidation may be similar to phosphorylation, having the capacity to both inhibit and stimulate kinase activity, depending on the site of modification (42).

In this study, we analysed the impact of *Spn*-derived H_2_O_2_ on kinase activity and identified differentially regulated kinases in H441 lung cells infected with *SpnWT*, *Spn*Δ*lctO*Δ*spxB* or *Spn*Δ*ply* as compared to non-infected cells. The deletion of the two main H_2_O_2_ producing enzymes SpxB and LctO in *Spn* decreases the H_2_O_2_ levels to 3% of that measured in *SpnWT* (11). Our results show that infection of H441 cells with *SpnWT* significantly downregulated a large number of kinases. Kinase activity profiles of *Spn*Δ*lctO*Δ*spxB-* vs. *SpnWT*-infected cells were significantly different and revealed higher activity of several kinases in the absence of H_2_O_2_ generation in *Spn*Δ*lctO*Δ*spxB*, including Akt, Lmr1, FGFR1, FLT3, Brk, CK1, AMPKα1, PKG1, PKG2, PKAα and PRKX (**Figure 3A**). Further kinases (Lck, FRK, Src Fyn, BLK, Hck, Yes, CDK1, CDK2, ERK1, ERK2, ERK5, p38-δ, p38-γ, AlphaK1 and SgK307) were found to be moderately dysregulated in *Spn*Δ*lctO*Δ*spxB-* vs. non-infected cells (**Figure 3B**), likely due to H_2_O_2_-independent effects. The similar intensities of peptide phosphorylation in cells infected with *SpnWT* and *Spn*Δ*ply* suggest that the previously described reduction of Ply release in the *Spn*Δ*lctO*Δ*spxB* mutant is not the main cause for the distinct kinome profile of *Spn*Δ*lctO*Δ*spxB*-infected cells (**Figures 2A–D**), thus substantiating the important role of pneumococcal H_2_O_2_ (24, 25). We selected two of the top ranked significantly dysregulated kinases identified during infection with *SpnWT* - Akt kinase and the Src family kinase Lck- for further Western blotting analysis.

Our results show that infection of H441 cells with the H_2_O_2_-producing strain *SpnWT* decreases Akt protein expression as well as Akt phosphorylation, assessed as the p-Akt/Akt ratio in H441 cells. In contrast, Akt protein levels and p-Akt/Akt ratio in *Spn*Δ*lctO*Δ*spxB* infected cells as well as non-infected cells are significantly higher (**Figures 5A, B**). The reduced phosphorylation of Akt during infection of H441 cells with *SpnWT* that we observed correlates well with the findings previously reported with *Spn* infection in the A549 epithelial cell line, which also demonstrated reduced Akt activity (43). Noteworthy, we demonstrate that external addition of H_2_O_2_ to cells infected with *Spn*Δ*lctO*Δ*spxB* decreases the phospho-Akt and total Akt levels comparable to cells infected with *SpnWT* (**Figure 8A**). These results suggest that pneumococcal*-*derived H_2_O_2_ reduces the total Akt and p-Akt levels.

The Src family kinase Lck was reported to be inactivated by phosphorylation of inhibitory tyrosine residue (Tyr-505) in the carboxyl-terminal region (32, 34). Lck phosphorylation at Tyr-505 stabilizes Lck in a biologically inactive conformation, as it associates intramolecularly with the SH2 domain in the amino-terminal half of the protein (33). Our results indicate that infection of H441 cells with the H_2_O_2_-deficient *Spn*Δ*lctO*Δ*spxB* strain, but not with H_2_O_2_-producing strains *SpnWT* and *Spn*Δ*ply*, increases phosphorylation of Lck at Tyr-505 (**Figures 7A, B**). These results suggest an H_2_O_2_-dependent activation of Lck in the presence of pneumococcal-derived H_2_O_2_, albeit in an indirect manner, i.e. through inhibition of inhibitory phosphorylation. The impact of H_2_O_2_ on Lck was substantiated, as also demonstrated for Akt, by addition of exogenous H_2_O_2_ to *Spn*Δ*lctO*Δ*spxB*-infected cells. The addition of H_2_O_2_ to *Spn*Δ*lctO*Δ*spxB*-infected cells led to a reduction in Lck phosphorylation, similar as what was observed upon infection with *SpnWT* (**Figures 8B–D**). These results suggest that reduced Lck phosphorylation at Tyr-505 depends on H_2_O_2_.

In summary, we provide direct evidence that pneumococcal-derived H_2_O_2_ has the capacity to modify host kinase activity in the course of *Spn* infection. We describe for the first time pneumococcal H_2_O_2_-specific downregulation of the activity of Akt kinase and activation of Lck. The exact mechanisms and signal pathways responsible for these effects require further study.

## Data availability statement

The original contributions presented in the study are included in the article/**Supplementary Material**. Further inquiries can be directed to the corresponding author.

## Ethics statement

Ethical approval was not required for the studies on humans in accordance with the local legislation and institutional requirements because only commercially available established cell lines were used. Ethical approval was not required for the studies on animals in accordance with the local legislation and institutional requirements because only commercially available established cell lines were used.

## Author contributions

JaB: Formal analysis, Methodology, Validation, Visualization, Writing – original draft. AW: Project administration, Supervision, Writing – original draft, Data curation, Formal analysis, Software, Visualization. JuB: Software, Methodology, Writing – review & editing. RS: Software, Writing – review & editing, Resources. TH: Resources, Data curation, Writing – original draft. RL: Resources, Writing – original draft, Formal analysis, Writing – review & editing. MM: Resources, Writing – original draft, Writing – review & editing, Conceptualization, Funding acquisition, Investigation, Project administration, Supervision.
